# Analysis of potential protective effects of caffeic acid phenethyl ester against gentamicin ototoxicity: An experimental study

**DOI:** 10.22038/IJBMS.2022.60794.13467

**Published:** 2022-01

**Authors:** Fuat Aydemir, Cagatay Han Ulku, Cigdem Elmas, Cemile Merve Seymen

**Affiliations:** 1 Department of Otorhinolaryngology, Kulu State Hospital, Konya, Turkey; 2 Department of Otorhinolaryngology, Necmettin Erbakan University Meram Faculty of Medicine, Konya, Turkey; 3 Department of Histology and Embryology, Gazi University Faculty of Medicine, Ankara, Turkey

**Keywords:** Aminoglycoside, Anti-oxidant, Caffeic acid phenethyl ester, Gentamicin, Ototoxicity

## Abstract

**Objective(s):**

In this study, it is aimed to investigate the potential protective effect of caffeic acid phenethyl ester (CAPE) on ototoxicity caused by gentamicin in a rat model.

**Materials and Methods:**

Thirty Wistar albino rats were divided into 3 groups. Group I was selected as the control group. Gentamicin was administered intraperitoneally in group II, gentamicin and CAPE in group III. Audiological assessment was performed by the distortion product otoacoustic emission (DPOAE) and auditory brainstem response (ABR) measurements before and after treatment of each group. At the end of the study all rats were decapitated, cochlea was removed and electron microscopic examination was performed.

**Results:**

In group II post-treatment DPOAE levels were found to be lower than pretreatment DPOAE levels (*P*<0.05). However, in group III, there is no significant difference between pre- and post-treatment DPOAE levels (*P*>0.05). Except for Group I, ABR thresholds increased after the procedure and this increase was statistically significant (*P*<0.0001). According to histological examination by transmission electron microscopy, CAPE has a cellular protective effect against gentamicin ototoxicity.

**Conclusion:**

CAPE may ameliorate hearing deterioration caused by gentamicin ototoxicity and protect the cochlear cells from apoptosis due to the strong antioxidant effect.

## Introduction

Ototoxicity is defined as damage caused by the use of some medicines and chemicals and usually occurs permanently in the inner ear. Agents that can cause ototoxicity are aminoglycosides, antineoplastic drugs (cisplatin/carboplatin), loop diuretics, quinine, and salicylates. Aminoglycoside antibiotics are used clinically in various infectious diseases caused by gram-negative bacteria. However, the clinical use of aminoglycoside antibiotics is limited due to serious side effects and particularly ototoxicity which can result in hearing loss, tinnitus, and vestibular disorders (1). Despite all this, they are very inexpensive to produce, which is an important reason for preference for the economically weak population in developing countries (2). Although there are studies in the literature about the protective efficacy of many agents against aminoglycoside ototoxicity, there is still no accepted treatment protocol.

The frequency of ototoxicity in patients who were treated with gentamicin varies between 2% and 25% (1). The gentamicin-induced ototoxicity is dose-dependent and the probability of improvement is very low when the hearing was damaged. The most common cause of gentamicin-induced hearing impairment is loss or dysfunction of hairy cells with special sensory receptors that convert a mechanical stimulus into nerve impulses (3). 

Propolis, a honeybee product, is a resinous product obtained from different plant parts mixed with beeswax and bee salivary enzymes (4, 5). It has been used in traditional medicine for many years due to its anti-inflammatory, antiviral, antimitogenic, anticarcinogenic, immunomodulatory, and anti-oxidant effects (6). Caffeic acid phenethyl ester (CAPE) is the main component that mediates most of the beneficial effects attributed to propolis (7). CAPE administration in rat models has been shown to have protective effects against oxidative damage caused by different agents in various tissues such such as kidney, liver, heart, lung, brain, and neural structures (8-12). Also, CAPE has a strong neuroprotective activity (13). In the literature, studies on administration of CAPE against the toxic effects of aminoglycoside in the inner ear are limited.

In our study, we aimed to define a new approach for the prevention of gentamicin ototoxicity with CAPE and the treatment of ototoxicity.

## Materials and Methods

This study was approved by the Ethics Committee on Animal Experiment and Research of Necmettin Erbakan University (Decision Number: 2016-038). Animals were purchased from the Laboratory for Experimental Animals of Necmettin Erbakan University and the study was done at the Laboratory for Experimental Animals of Necmettin Erbakan University. All experimental procedures were performed according to the stated rules in the Guide for the Care and Use of Laboratory Animals. A checklist of working with laboratory animals approved by the Ministry of Health was used.

***Animals***

This study was performed on 30 healthy female Wistar albino rats weighing between 250 and 300 g. Rats were kept in an environment where free food and water were available, at temperature of 22 ± 2 °C, humidity of 45–65%, and 12 hr light-12 hr darkness cycle.

***Study***

The animals were randomly divided into three groups. In our study, by evaluating the condition of the middle ear with a microscopic examination (Carl Zeiss Surgical GmbH-Strasse 22), rats without the presence of any debris and/or earwax and found to have normal eardrums were included in the study. 

Group I (n=10) (control): A single dose of 2 ml/kg of saline was administered intraperitoneally for 21 days.

Group II (n=10) (gentamicin): 120 mg/kg gentamicin (Genta ampul, I.E. Ulugay) was administered intraperitoneally for 14 days. Then 2 ml/kg of saline was administered intraperitoneally for 7 more days. Three rats died during the study.

Group III (n=10) (gentamicin plus CAPE): 10 µmol/kg CAPE (Sigma-Aldrich Co LLC, St Louis, MO, USA) was administered intraperitoneally for 14 days with gentamicin (120 mg/ kg/day-14 days) and then CAPE was administered for 7 more days. 3 rats died during the study.

The rats were sedated using an intramuscular combination of xylazine 10 mg/kg (Rompun, Bayer, Turkey) and ketamine hydrochloride 50 mg/kg (Ketalar, Eczacibasi, Turkey) before distortion product otoacoustic emission (DPOAE) and auditory brainstem response (ABR) measurements and surgery.

***DPOAE test procedure***

DPOAE measurements were made with the Otodynamics Echoport ILO292 USB II device by using the premature newborn probe. At the beginning of the study and after 3 days of observation at the end of the study, the DPOAE test was applied to both ears of all rats. The ratio of frequencies f_2_ and f_1_ (f_2_/f_1_) was held to be 1.22. The stimulus intensity was taken as L1 for frequency f_1_ and L2 for frequency f_2_ and the difference between levels L1- L2 was kept at 10 dB SPL (L1 = 65 dB SPL, L2 = 55 dB SPL). DPOAEs were measured at 2f_1_-f_2_ frequency. The values that were 3 dB above the noise threshold of the DPOAE amplitudes were considered significant. The signal-to-noise ratios (SNR) at 1000, 1400, 2000, 2800, 4000, 6000, and 8000 Hz were recorded in the geometric averages of f_1_ and f_2_. SNRs were used in evaluating DPOAE results.

***ABR test procedure***

ABR measurements were performed on all rats bilaterally, pre- and post-study after 3 days of observation. ABR measurements were made with the Medelec Synergy ABR Device. Subdermal needles (Reusable Tip300 Insert Phones, 13 mm, Natus / 041-704000) and premature newborn ear probes (Eartips for use with Intra Auricular Headset and Ear Phone Type: Premature Natus/51023) were used for ABR measurements. The negative electrode was placed on the mastoid, the positive electrode on the test side, and the ground electrode on the opposite side to the mastoid. The suitability of the electrodes was checked with an impedance meter. ABR potentials were measured using 11.00 rate click stimuli, 10 msec measurement time, 100–1500 Hz filtering rarefaction polarity. Averages of 1500 click stimuli were received. The test was started with 70 dB SPL, the threshold was obtained by reducing the SPL in 10 dB steps, then the fine-tuning level in 5 dB steps up and down to identify the ABR pattern could be recognized. The ABR threshold was defined as the lowest level of intensity at which a V wave was observed.

***TEM***

The cochlea was taken into an ethylenediaminetetraacetic acid (EDTA) 10% decalcification solution containing glutaraldehyde 2% for approximately 15 days to complete the decalcification process and fixation. After the decalcification procedures, they were taken to the solution of 4% glutaraldehyde to wait for a while to complete the fixation. Then, tissue samples were placed in 1% osmium tetraoxide for one hour, followed by fixation and staining. Afterward, samples were dehydrated with alcohol series, and tissues were placed in propylene oxide for 30 min, followed by a 30 min waiting period in embedding material, enabling tissue passage into embedding material. After this, tissues taken into embedding material were placed into a rotator at room temperature for two hours, then placed into a 40 ºC oven for another two hours. Finally, tissues were embedded into horizontal embedding blocks within the same mixture (14). Thin sections were cut with a Leica EMUC7 ultramicrotome using a diamond knife (Leica EMUC7, Hernalser Hauptstrasse, Germany), mounted on a copper grid, and stained with 2% uranyl acetate and lead citrate. The grids were examined under a Carl Zeiss EVO LS 10 TEM-SEM microscope (Germany).

***Statistical analysis***

Statistical analyses were carried out using SPSS 22.0 (SPSS Inc, Chicago, IL, USA). The normal distribution of the data was confirmed by Kolmogorov-Smirnov test and histograms. Paired t-test was used to evaluate whether there was a difference between the SNR levels and ABR thresholds measured before and after treatment in each group. The differences in SNR levels and ABR thresholds between the groups were evaluated with the ANOVA test using the Tukey test as a *post-hoc* test. *P*<0.05 was accepted for statistical significance.

## Results

***DPOAE test results***

Pretreatment DPOAE SNR levels were compared between groups at all frequencies, and there were no significant differences (*P*>0.05).

**Figure 1:**
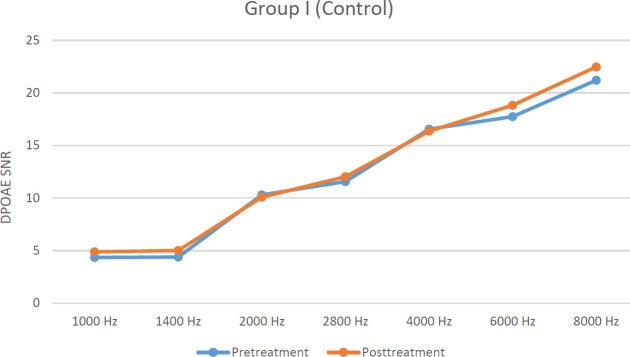
Graph demonstrating pre-and posttreatment DPOAE SNR levels in group I (Hz: Hertz, SNR: Signal to noise ratio of rats in control group)

**Figure 2:**
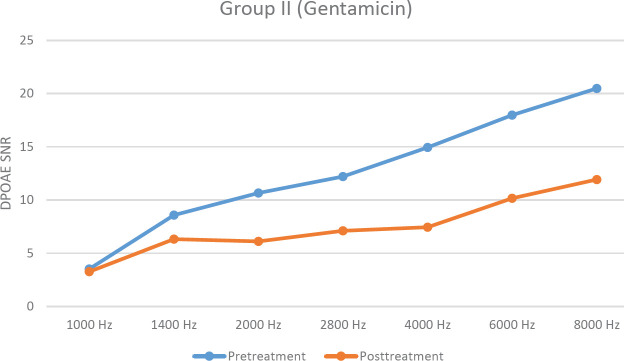
Graph demonstrating pre-and posttreatment DPOAE SNR levels in group II (Hz: Hertz, SNR: Signal to noise ratio of rats in gentamicin group)

**Figure 3:**
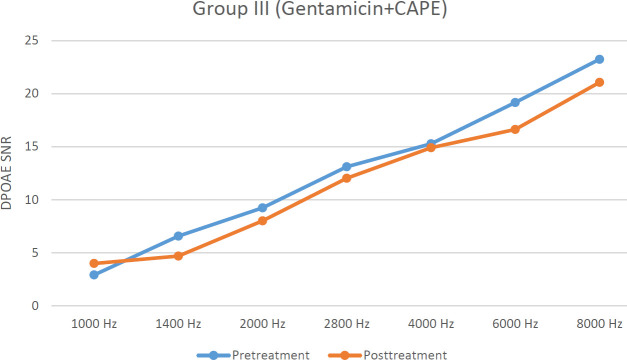
Graph demonstrating pre-and posttreatment DPOAE SNR levels in group III (Hz: Hertz, SNR: Signal to noise ratio, CAPE: Caffeic acid phenethyl ester)

There were no significant differences between pre- and post-treatment DPOAE SNR levels at any frequencies for group I (*P*>0.05). In group II, post-treatment DPOAE SNR levels were significantly lower in all frequencies except 1 and 1.4 kHz (*P*<0.05). There were no significant differences between pre- and post-treatment DPOAE SNR levels at 1, 1.4, 2, 2.8, and 6 kHz in group III (*P*>0.05). Pre- and post-treatment DPOAE SNR levels are shown in Figures 1, 2, and 3.

When post-treatment DPOAE SNR levels were compared among groups, for groups I and II, DPOAE SNR levels were significantly higher in group I at 2.8, 4, 6, and 8 kHz (*P*<0.05). On the other hand for groups I and III, there were no significant differences at any frequencies except 1 and 1.4 kHz (*P*>0.05). 

***ABR test results***

No statistically significant differences in the ABR threshold values were found between the groups’ pretreatment (*P*>0.05). The ABR threshold values of all groups pre- and post-treatment are shown in Table 1. It was observed that the ABR threshold values increased after the procedure for all groups and this increase was statistically significant (*P*<0.0001). The threshold values were found to be high in group II when group II and other groups were compared, but this was not statistically significant (*P*>0.05).

***TEM observation***

**Figure 4:**
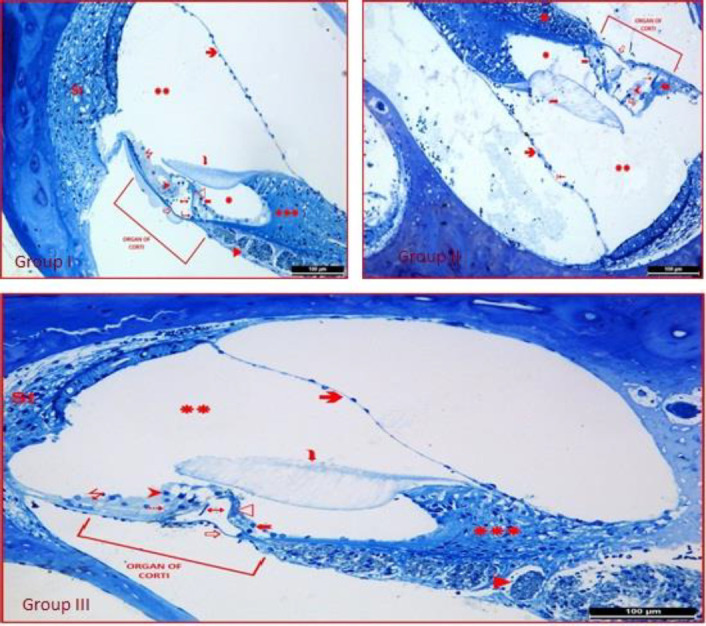
Semi-thin sections of all groups.: Reissner’s membrane (Vestibular membrane),: Basilar membrane, : Tectorial membrane, : Ductus cochlearis, : Spiral limbus,: Bundles of afferent and efferent nerve fibers,: Inner hair cells, : Outer hair cells, : Tunnel of Corti,: Inner phalangeal cells, : Boettcher-Claudius-Hensen cells, : Epithelial shedding, : Outer hair cells with pyknotic nuclei : Necrotic cells : Deiters (Outer phalangeal) cells, St: Stria vascularis (Toluidin blue x200)

In the examinations made on semi-thin sections prepared for TEM research; vestibular membrane (Reissner membrane), basilar membrane, and tectorial membrane were observed with their normal structures in group I. The inner spiral sulcus and cochlear duct, spiral limbus, and bundles of afferent and efferent nerve fibers were distinguished by their normal structure. The inner and outer hair cells and Corti tunnel of the Corti organ were observed with normal histological structures. Stria vascularis lining the basal turn of the cochlear duct was normal. By examinations made on the semi-thin sections for group II, the organ of Corti showed significant degenerative findings. It was observed that the basilar membrane had a degenerative appearance, the tectorial membrane lost homogeneous structure, gained fibrillar structure, and the tectorial membrane lost connection with the inner and outer hair cells. Many inner and outer hair cells decreased numerically relatively. In group III, basilar and vestibular membranes and stria vascularis were distinguished by normal structures. It was observed that the protective effect of CAPE administration was significantly strong. The tunnel of Corti was normal. Outer hair cells, Deiters (outer phalangeal) cells, and their relationship with the tectorial membrane were normal (Figure 4).

**Table 1:** Comparison of ABR thresholds pretreatment and posttreatment within – group for all groups

**Group**	**Pretreatment (dB nHL)**	** Posttreatment (dB nHL)**	***p***
Group I	17,5 ± 8,89	16,5 ± 6,25	0,775
Group II	14 ± 7,74	43,57 ± 6,9	<0.001^*^
Group III	15,5 ± 11,41	32,85 ± 10,35	0.015

*Statistical analysis is performed with Paired T-Test *P*<0.001

## Discussion

Ototoxicity is a clinical condition due to damaged inner ear structures secondary to drugs and therapeutic agents and leads to such symptoms as balance disorder and tinnitus (2). Ototoxicity may be both temporary and permanent. Many agents cause ototoxicity. The main ones are aminoglycosides, antineoplastic drugs (cisplatin/carboplatin), loop diuretics, quinine, and salicylates. These may have cochleotoxic, vestibulotoxic, or both effects. 

Gentamicin damages the outer hair cells, starting from the basal region of the cochlea, then this damage proceeds to the apical. Therefore, hearing loss starts at high frequencies and then progresses to low frequencies. Damage to outer hair cells can be evaluated with DPOAE. The ABR test is an objective test used in evaluating hearing pathways from proximal to distal (15). In this study, DPOAE and ABR electrophysiological tests were used for auditory evaluation. Fetoni *et al*. (16) reported an increase in ABR threshold shift at high and medium frequencies and a high degree (60%) of outer hair cell loss in the basal and middle turns in the guinea pigs after gentamicin injection. In our study, posttreatment DPOAE values especially in high frequencies were lower than pretreatment DPOAE values, and posttreatment ABR thresholds were higher than initially in the gentamicin group. It has been demonstrated that ototoxicity occurred with the administration of 120 mg/kg gentamicin (intraperitoneal) once a day for 15 days. According to this study gentamicin doses of less than 120 mg/kg are not enough to cause ototoxicity (17). Therefore, 120 mg/kg gentamicin was administered intraperitoneally for 14 days in this study and ototoxicity occurred according to DPOAE SNR levels and ABR thresholds.

In our study, as a result of electron microscopic examination, it was determined that gentamycin which was administered as an intraperitoneal injection, caused serious degeneration in the inner ear and organ of Corti. According to DPOAE, ABR, and histopathological results, ototoxicity occurred with gentamicin.

Clinically, aminoglycoside drugs enter the inner ear through systemic and topical pathways. In the systemic pathway, the drug enters the inner ear through the stria vascularis while the drug enters the inner ear through the round window in the topical pathways (18). Free oxygen radicals are thought to be the main cause of aminoglycoside ototoxicity. Aminoglycosides are non-lipophilic and enter into the hairy cell through mechano-electric transducer channels. Then the formed aminoglycoside-iron complex reacts with electron donors such as free oxygen radicals consisting of arachidonic acid metabolism. As a result of pathways activated by free oxygen radicals, the cell undergoes apoptosis. Free oxygen radicals play a key role here (19, 20). In many studies, it is emphasized that ototoxicity occurs due to the formation of reactive oxygen metabolites in the inner ear. Therefore, many studies are aiming to prevent aminoglycoside induced ototoxicity by using various anti-oxidant substances such as iron chelator (deferoxamine), D-methionine, dexamethasone, coenzyme Q10, dihydroxybenzoate, salicylate, N-acetylcysteine, and memantine (2, 16, 17, 21-24). 

Oxidative stress is neutralized by anti-oxidants in physiological conditions. Anti-oxidants protect the cell against the undesirable effects of drugs, carcinogens, and toxic substances. Anti-oxidants inhibit lipid peroxidation by blocking the peroxidation chain reaction and scavenging reactive oxygen species. To minimize or eliminate the damage caused by free oxygen radicals, it is necessary to prevent the increase of free oxygen radicals, and trigger biochemical events or use anti-oxidants. Anti-oxidant use is the most important and popular method among them (25). Although there are many studies done to prevent gentamicin ototoxicity, there is currently no agent accepted for routine use in ototoxicity. In this study, we investigated the protective effect of caffeic acid phenethyl ester, which is a powerful anti-oxidant, against gentamicin ototoxicity.

CAPE, a well-known component of the natural honey bee product propolis, has been used in medicine for centuries due to its anti-inflammatory, anti-oxidant, and antineoplastic effects (26). It reduces pro-inflammatory cytokines and inflammatory mediators by inhibiting nuclear factor kappa-B (NF- κB) transcription (27). CAPE has antimitogenic, anticarcinogenic, anti-inflammatory, and immunomodulating effects *in vitro* (26). The reason why CAPE passes membranes easily is two hydroxyl (-OH) groups that it carries with the phenyl and polyhydrocarbon chain. These two hydroxyl groups give the molecule strong anti-oxidant properties. 

In our study, according to our DPOAE results, it was shown that CAPE had a protective effect against gentamicin ototoxicity. Although ABR thresholds were lower in group III compared with group II, this decrease was not statistically significant. In a study, the effect of CAPE on cisplatin ototoxicity in rats was investigated and it was reported that CAPE attenuated hearing loss according to DPOAE findings. Besides, plasma xanthine oxidase activity was increased more in the cisplatin group, but CAPE decreased xanthine oxidase activity (28). Bakir *et al*. (29) evaluated the anti-oxidant properties of CAPE for the prevention or reduction of ototoxicity caused by the use of an aminoglycoside in rats. In the immunohistochemical and histopathological examinations performed in addition to hearing tests, cochlear hair cells were not disrupted in the group treated with CAPE. These results show that CAPE has protective effects against reactive oxygen species caused by oxidative pathways in the inner ear. In another study, the protective effect of CAPE against hairy cell damage caused by neomycin in zebrafish was examined and it was reported that apoptosis and apoptotic cell deaths induced by neomycin could be prevented with CAPE (27). In our study, as a result of electron microscopic examination for group III, it was determined that administration of CAPE had a good protective effect on the inner ear and the Corti organ. 

There are some limitations in our study. First, the number of animals in each group was small. Secondly, we did not perform the dose comparison for the CAPE effect. In a study, it was stated that CAPE at a concentration of 10 µmol/kg completely inhibited reactive oxygen species (30). In the light of this information, we used a dose of 10 µmol/kg CAPE in our study. We did not investigate the effect of CAPE on the antimicrobial activity of gentamicin. This is another limitation of our study. Further studies are required to evaluate the protective effect of CAPE against gentamicin-induced ototoxicity and the efficacy of CAPE in the antimicrobial activity of gentamicin.

## Conclusion

Although many studies have been done to prevent ototoxicity, there is still no accepted treatment protocol. In this study, CAPE has been shown to have a protective effect on gentamicin-induced ototoxicity with both DPOAE results and TEM. We conclude that this protective effect is due to its strong anti-oxidant properties and may prevent or ameliorate the ototoxic effect of gentamicin. It is the first study in the literature to investigate the effect of caffeic acid phenethyl ester on gentamicin-induced ototoxicity using both electrophysiological tests and electron microscopy. However, further studies that combine more definitive electrophysiological and histomorphological examinations are needed to evaluate the protective effect of CAPE on gentamicin-induced ototoxicity. 

## Authors’ Contributions

CHU Supervised, conceived the original idea, verified the analytical methods, and checked the whole procedure and paper. CE and CMS Performed the histopathological examination and analyzed the results. FA Processed the data, performed experiments, and wrote the paper. All authors have read and approved the paper.

## Conflicts of Interest

The authors declare that no conflict of interest exists.
